# Role of the XPA protein in the NER pathway: A perspective on the function of structural disorder in macromolecular assembly

**DOI:** 10.1016/j.csbj.2015.11.007

**Published:** 2015-12-08

**Authors:** Elisa Fadda

**Affiliations:** Department of Chemistry, Maynooth University, Maynooth, Kildare, Ireland

**Keywords:** Structurally disordered protein domains, Nucleotide Excision Repair (XPA), NER pre-incision complex, Beads-on-a-string multiprotein complex, XPA, RPA, ERCC1-XPF, Conformational selection, Molecular recognition

## Abstract

Lack of structure is often an essential functional feature of protein domains. The coordination of macromolecular assemblies in DNA repair pathways is yet another task disordered protein regions are highly implicated in. Here I review the available experimental and computational data and within this context discuss the functional role of structure and disorder in one of the essential scaffolding proteins in the nucleotide excision repair (NER) pathway, namely *Xeroderma pigmentosum* complementation group A (XPA). From the analysis of the current knowledge, in addition to protein–protein docking and secondary structure prediction results presented for the first time herein, a mechanistic framework emerges, where XPA builds the NER pre-incision complex in a modular fashion, as “beads on a string”, where the protein–protein interaction “beads”, or modules, are interconnected by disordered link regions. This architecture is ideal to avoid the expected steric hindrance constraints of the DNA expanded bubble. Finally, the role of the XPA structural disorder in binding affinity modulation and in the sequential binding of NER core factors in the pre-incision complex is also discussed.

## 1 Introduction

The regulation of molecular function by structurally disordered protein domains is currently one of the hottest topics in structural and computational biology [1], [2], [3], [4]. Over 40% of proteins coded by the human genome contain structurally disordered regions [3], [5], usually involved in regulatory and signaling pathways [4]. Notably, the dysregulation of poorly structured protein domains is also linked to diseases [5], making these regions an interesting, but very challenging, target for drug design. One of the main advantages of the lack of structure in protein domains is in the flexibility it confers when the protein interacts with multiple counterparts. DNA repair pathways hinge on complicated interaction networks that involve many different proteins. Some of these proteins need to assemble into large complexes to perform their specific tasks and these associations are often reversible and transient. Structural disorder is instrumental toward the coordination of these reversible and transient networks [6], thus crucial toward the effectiveness of DNA repair. *Xeroderma pigmentosum* complementation group A (XPA) is an essential protein in the nucleotide excision repair (NER) pathway, the main DNA repair pathway responsible for the excision of bulky DNA lesions in both eukaryotic and prokaryotic cells. NER targets lesions caused by environmental mutagens, such UV light and polycyclic aromatic hydrocarbons, or by alkylating agents, such as platinum-based chemotherapeutics [7]. Enhanced expression levels of NER core factors have been directly linked to clinical resistance to platinum chemotherapy [8], [9], making the NER pathway and the proteins involved in it very interesting targets in cancer research [10], [11], [12], [13]. One of the key roles of XPA is to coordinate the assembly of other NER core factors around the DNA damage site [14], [15], [16], [17] before lesion excision. The mechanistic details of this process are not clear, especially because of the lack of structural information available on the scaffolding protein, XPA, which is largely disordered. In this work I will summarize and discuss the structural, mutagenesis, and computational data available to date to produce an overall 3D mechanistic model of the assembly of the NER pre-incision complex. This discussion will help to provide a clearer understanding of the essential function of the structural disorder of XPA as a flexible scaffolding protein and its mechanistic role in the NER pathway. In the following sections, the author will 1) outline the main steps of the NER pathway up to the damaged oligonucleotide excision, 2) discuss the XPA sequence within the context of its secondary structure and protein–protein and protein–DNA interaction regions, 3) describe the structure or, when a complete structure is not available, define a 3D model for the 3 essential XPA–protein complexes (modules) that make the pre-incision complex, and finally 4) discuss the functional and mechanistic role of a poorly structured XPA scaffold in the modular assembly, as “beads on a string”, of NER core factors.

## 2 The nucleotide excision repair (NER) pathway

**Fig. 1:**
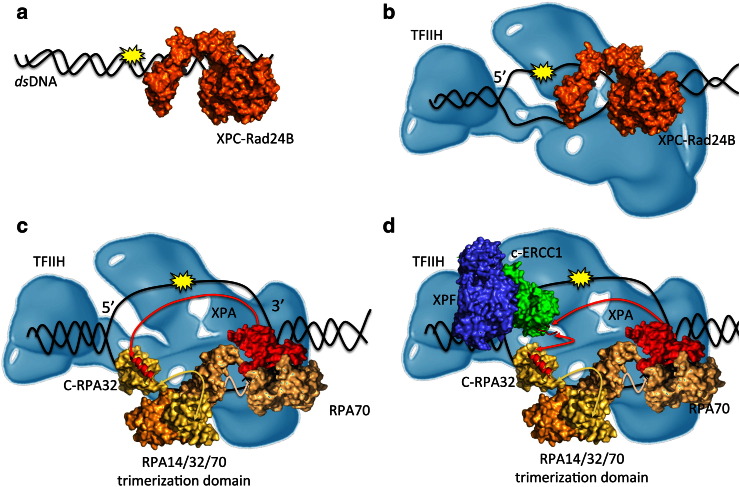
Schematic representation of the damage recognition to excision steps in the GG-NER pathway. (Panel a) The XPC-RAD23B heterodimer, shown in orange and represented by the structure of the yeast ortholog Rad4 (PDBid 4YIR), is responsible for the detection of bulky DNA adducts and initiation of the GG-NER pathway. (Panel b) XPC–RAD23B recruits the multidomain complex TFIIH, shown in light blue and adapted from ref. [85]. TFIIH includes two helicases, namely XPB and XPD, which coordinate the duplex opening. (Panel c) XPA and RPA, shown in red and tan, respectively, are recruited to the lesion site possibly as a pre-formed complex. This interaction causes the release of XPC–RAD32B. The complex between the 3 RPA domains and the ssDNA covers a 30 nucleotides stretch, corresponding to the full length of the DNA bubble. In this panel the structures representing the RPA32 C-terminal domain, the RPA trimerization domain, and the RPA70 *ss*DNA binding domain correspond to the PDBids, 1DPU, 1L10, and 1JMC, respectively. The complex between the XPA (1XPA), the *ss*DNA, and RPA70 (1JMC) has been obtained by protein–protein docking and is discussed in the text. The red line connecting XPA to the RPA32 C-terminal domain represents the intrinsically disordered XPA N-terminal 97 aa disordered linker. (Panel d) The binding of XPA promotes the recruitment of the ERCC1-XPF endonuclease to the 5′ side of the lesion. The structures used to build a model of the ERCC1-XPF complex by alignment have PDBids 2A1I, 2A1J, 2BGW, and 2JNW, which represent the central domain of human ERCC1, the C-terminal domains of human XPF and ERCC1, the XPF from *Aeropyrum pernix*, and the central domain of human ERCC1 in complex with XPA_67–80_, respectively.

Although significantly different in their chemistry, all NER-targeted lesions have a common structural trait, namely a severe bend in the DNA that destabilizes the double helix [18]. Such DNA damage can be detected either when it stalls the RNA polymerase, which initiates the so-called transcription-coupled NER (TC-NER) pathway, or independently of transcription, through the action of specific damage sensing proteins that initiate the global genomic NER (GG-NER) pathway [18]. TC-NER and GG-NER differ significantly only in this damage recognition step. Eukaryotic NER involves the work of over 30 proteins [19], implicated in a sequential series of actions that can be summarized as follows: 1) DNA damage recognition, 2) DNA unwinding, 3) 3′ and 5′ dual incision, 4) damaged oligonucleotide removal, 5) gap-filling, and finally, 6) ligation [18], [20]. Specific NER proteins carry out these steps through the progressive and coordinated formation of multi-protein assemblies [18], [21], [22], [23]. Shown in Table 1 are the core NER factors responsible for damage recognition and excision in eukaryotic cells, namely *Xeroderma pigmentosum* complementation group C (XPC) in complex with RAD23B, replication protein A (RPA), XPA, the transcription factor II H (TFIIH) complex, XPG, and the excision repair cross-complementation group 1 (ERCC1) in complex with XPF [20]. The prefix “XP” shared by 7 NER core factors, from XPA to XPG, derives from their identification though genetic complementation studies of the human DNA repair disease *Xeroderma pigmentosum*[24]. XPC–RAD23B and ERCC1–XPF are stable heterodimeric complexes [25], [26], [27], [28], [29]. RPA is an *ss*DNA-binding multimodular heterotrimer composed by 3 subunits, namely RPA32, RPA14, and RPA70 [30], where the numbering refers to their respective molecular weights. Finally, TFIIH is a large multi-domain complex, containing 6 subunits, which include the two helicases XPB and XPD [31], [32], and the cyclin-dependent kinase (CDK)-activating complex (CAK) [32]. The main steps leading to the damage excision in the GG-NER pathway are summarized in Fig. 1. The XPC–RAD23B complex is responsible for initiating the GG-NER pathway by detecting helical distortions caused by bulky DNA adducts [18], [20], [31]. Notably, compelling evidence shows also that XPA binds specifically distorted DNA helices and could be involved in the recognition of bulky DNA lesions [33], [34]. Furthermore, recent structural work on Rad14, the yeast homolog of human XPA, suggests that the XPA homodimer can be involved in the DNA damage detection [34]. Indeed, XPA dimerization has been previously reported, but its functional role in NER was not clear [35], [36]. As shown Fig. 1, panel b, the XPC–RAD23B recruits the TFIIH multidomain helicase to the damage site [22]. The two helicase subunits of TFIIH, namely XPB and XPD, unwind the double helix, exposing a 30 base-pair long *ss*DNA stretch carrying the lesion, a structure known as DNA bubble [19], [23]. The N-terminal domain of XPC also interacts with XPA [37], [38], [39], which could be recruited at this stage together with RPA, possibly as a pre-formed XPA–RPA complex [40], as shown in Fig. 1, panel c [41]. Park et al. [42] have shown that XPA interacts with TFIIH and that TFIIH may be involved in its recruitment, or in the recruitment of the XPA–RPA complex. Lastly, if an XPA homodimer (XPA_2_) is involved in damage recognition, it would be already located on the forming bubble, however the transition from XPA_2_ to the DNA-bound XPA–RPA complex is not clear as yet. The XPA–RPA interaction promotes the dissociation of the XPC–RAD23B from the damage site [43], which results in the dissociation of the XPC–RAD23B dimer [25]. It is at this stage, represented schematically in Fig. 1, panel d, that the XPA serves its most important role as a scaffold, by coordinating the multi-protein assembly and the docking of the 5′ specific endonuclease to the *ss* to *ds*DNA Y junction [15], [17], [18], [19], [44]. Whether XPA binds the *ss*DNA to *ds*DNA junction on the 5′ side [38] or the 3′ side [19], [44], [45] of the damage in the pre-incision complex has not been clearly established yet. However, based on structural constraints, the argument of XPA binding the Y DNA junction at the 3′ side seems more compelling. A possible arrangement of the NER factors around the DNA bubble, with the XPA positioned at the 3′ Y junction and the RPA on the undamaged oligonucleotide [19], [46], is represented schematically in Fig. 1, panel d. The 3′ specific XPG endonuclease (not shown) is most likely recruited to the damage site by TFIIH [18], [47], [48], to which it is associated [49]. The dual incision is initiated by the ERCC1–XPF at the 5′ side and then followed by the XPG at the 3′ side of the bubble [50]. The release of the damaged oligonucleotide, and of the TFIIH bound to it [51], leads to the unbinding of XPA and to the DNA re-synthesis and ligation [18].

## 3 XPA protein sequence and interactome

**Fig. 2:**
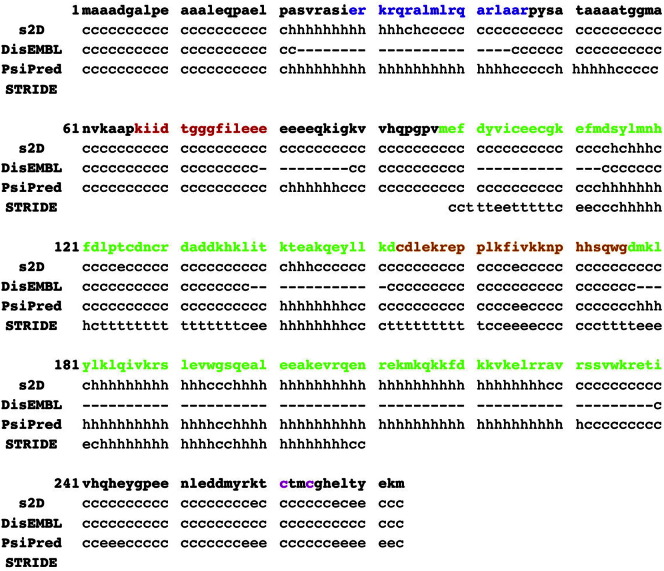
Sequence (NP_000371.1) of the full length XPA (*Homo sapiens*). Color coding is used to facilitate the mapping of the interaction motifs along the sequence, the RPA32 interaction motif is shown in blue, the ERCC1–XPF in red, the DNA in green, the RPA70 in orange, and the two Cys involved in the interaction with TFIIH in purple. Sequence-based secondary structure and disorder predictions obtained with the s2D [61], DisEMBL [63], and PsiPred [62] methods are also shown, together with the secondary structure assignments, obtained with STRIDE [60], based on the NMR structure of the XPA DNA binding domain (PDBid 1XPA). The one-letter code used to specify secondary structure motifs reads as follows, c (coil), t (turn), g (3–10 helix), h (α-helix) and e (β-sheet). Long stretches containing coils and/or turns indicate structural disorder. Predicted structured regions in DisEMBL are indicated with a dash.

**Fig. 3:**
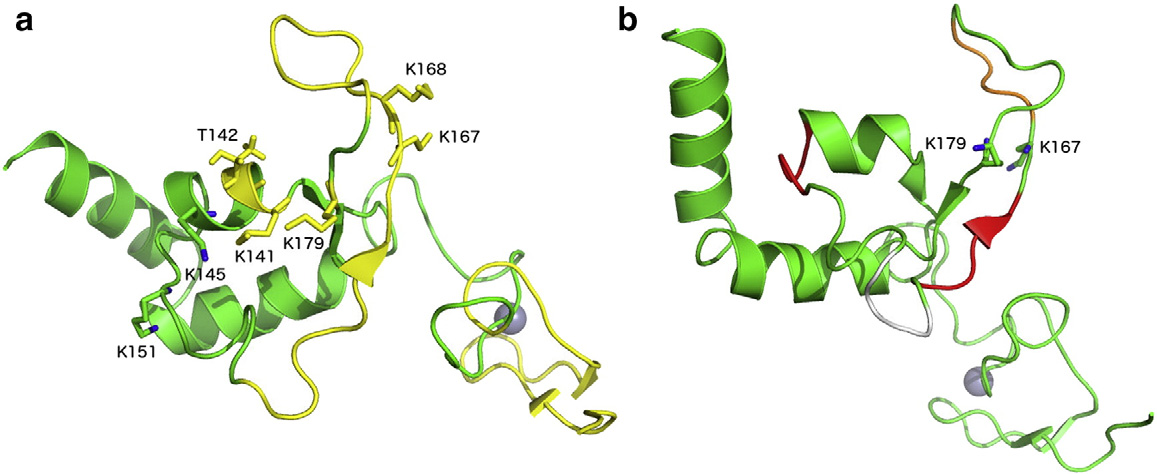
(Panel a) Structure of the XPA DNA-binding core (aa 98 to 210) represented with cartoon rendering. The regions implicated in RPA70 binding are shown in yellow, while the rest of the structure is shown in green. The Zn atom is shown as a gray sphere and the position of some of the key residues for DNA and RPA70 binding, are highlighted with sticks rendering. (Panel b) The 4 residue-long stretches implicated in RPA70 binding are shown in red, where the ΔLKFI stretch is in a β-sheet strand and the ΔEYLL is partly involved in a helical motif. The ΔKNPH stretch, which only moderately affects binding, is highlighted in orange and the ΔKREP, which does not affect binding, in white. Mutagenesis data from ref. [40].

As summarized in Table 1 and Fig. 1, XPA interacts directly with all NER core factors at the damage site, aside from XPG, functioning as a scaffold for the excision of the damaged oligonucleotide [15], [18]. The 273 residues (40 kDa) XPA protein contains a partially structured Zn-containing subdomain [52], [53], located between residues 98 to 219, and poorly structured C and N terminal tails [29], [54]. The XPA sequence and secondary structure assignments are shown in Fig. 2. The XPA Zn-containing core is responsible for binding both, the *ss*DNA to *ds*DNA junction, or Y junction [15], [55], and the RPA70 domain [17], [56]. Recent studies have shown that the XPA DNA binding domain extends beyond the known solution structure (PDBid 1XPA), up to residue 239; with the XPA_98–239_ construct found to bind DNA Y junctions with the same affinity as the full-length protein [15], [55]. The interaction with RPA70 was mapped onto the region between XPA residues 141 and 176 [17], [40], with the possible contribution of the Zn-containing subdomain [52], or of both [57], as shown in Fig. 3, panel a. The interaction with TFIIH involves a region included within the last 48 residues of the XPA C-terminal domain [42]. Although the XPA TFIIH-binding region has not been mapped in more details, an earlier study [42] has shown that the C261S and C264S XPA mutants are not able to bind TFIIH, see Fig. 2. The interactions with the RPA32 subunit and with the ERCC1–XPF 5′endonuclease involve different regions located in the structureless N-terminal tail of XPA [40], [54], [58]. As highlighted in Fig. 2, the stretch between residues 29 and 46 is involved in the interaction with RPA32 [29], while the stretch between residues 67 and 80 represents the minimal binding motif for the interaction with the ERCC1–XPF 5′ endonuclease [54], [58], [59]. As shown in Fig. 2, the protein–protein interaction regions of XPA are separated by long and disordered link regions. This architecture allows the NER proteins to associate to XPA to form a multiprotein complex with an overall “beads on a string” motif, where the beads can act cooperatively in a modular fashion. The identity, structure, and function of the different XPA–proteins modules in the pre-incision complex are discussed below.

## 4 Module 1: The XPA_98–219_–DNA–RPA70 complex

The only region of XPA structurally characterized is part of its DNA and RPA70 binding core, comprising residues 98 to 219 [17], [52], [53]. The XPA_98–219_ shown in Fig. 3, presents a 3 helix packing domain, located between residues 141 to 210, sided by a short β-sheet stretch and by poorly structured loop regions, which also include the Zn coordination site [52], [53]. STRIDE [60] secondary structure assignments based on the XPA_98–219_ solution NMR structure with PDBid 1XPA are shown in Fig. 2. Although no structural data is available at this time of the XPA in complex with *ss*DNA or with a Y DNA junction, recent studies have shown that two Lys, namely K168 and K179 are essential for DNA interaction [55], see Fig. 3, panel a. In the same study [55], a set of residues not included in the XPA_98–219_ NMR structure, and for which no structural data are available yet, have been also identified as important for DNA binding, namely K221, K222, K224, and K236. As shown in Fig. 2, according to sequence-based secondary structure prediction methods, namely s2D [61], and PsiPred [62], these Lys are located in a region, between amino acid (aa) 200 and 228, likely to fold into an α helix. The presence of a fourth helix in this region has also been proposed earlier, based on the interpretation of NMR data [15]. Accordingly, as also shown in Fig. 2, the sequence-based disorder prediction method DisEMBL [63] does not flag the 200 to 228 aa stretch as disordered.

**Fig. 4:**
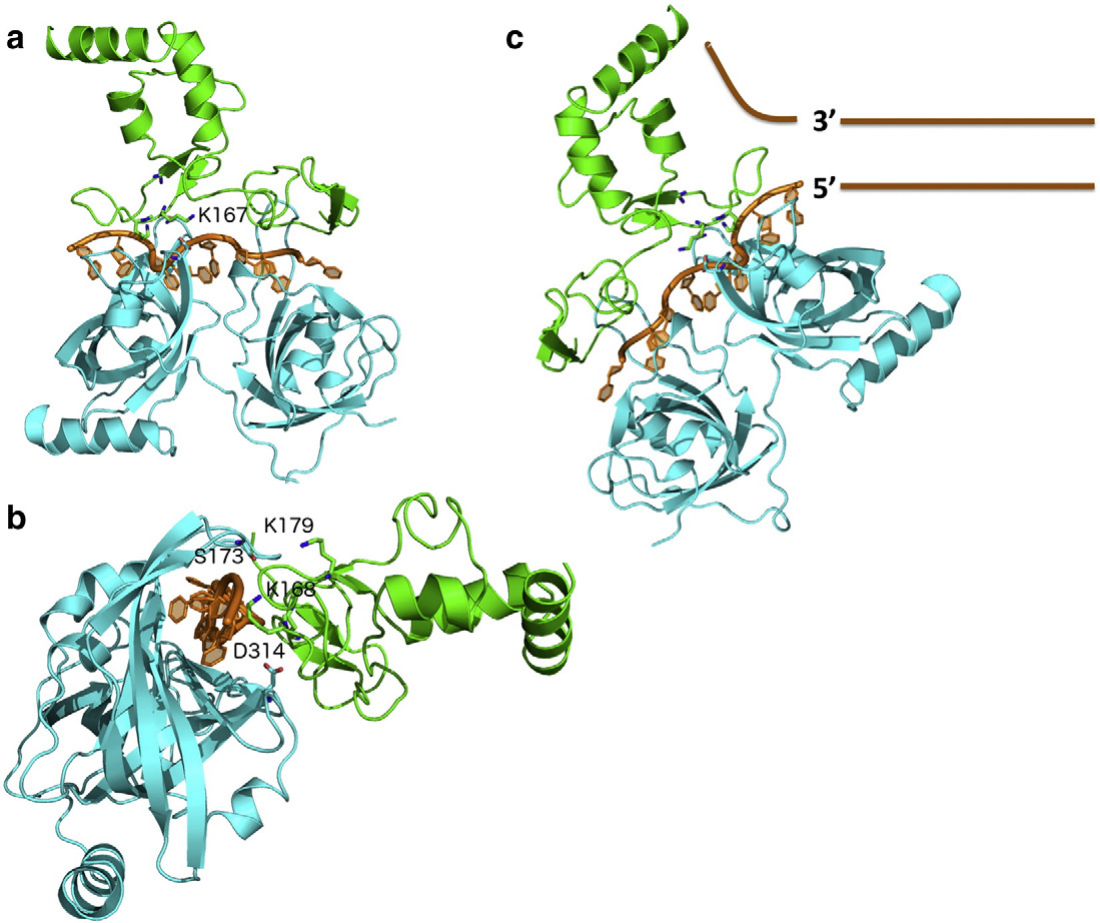
(Panel a) Highest scoring pose for the XPA_98–219_–ssDNA-RPA70 complex predicted by the ClusPro online tool [65], [66]. The XPA (1XPA PDBid) is shown in green, the RPA70 in complex with the ssDNA strand (1JMC PDBid) are shown in cyan and orange, respectively. (Panel b) Side view into the three-body XPA_98–219_–ssDNA-RPA70 complex showing RPA70 Ser 173 and Asp 314 as a possible contacts for XPA Lys 179 and Lys 167, respectively. (Panel c) Proposed organization of the three-body XPA_98–219_–*ss*DNA–RPA70 complex at the Y junction in the NER pre-incision complex.

Interactions with both, *ss*DNA and RPA70, take place around the same region within the XPA_98–219_ domain [17], [56], where the RPA70 binding region may extend to the Zn coordination site [52], [53]. K179 has been identified as a key residue for the binding of both, the *ss*DNA and RPA70 [17], [55], see Fig. 3, panel a, while adjacent residues, namely K167 and K168, have been identified as key residues for binding RPA70 and DNA, respectively [17], [45], [55]. NMR and mutagenesis data [17], [45] also highlight the involvement in the *ss*DNA binding of a number of other residues located on the α helix 1 of XPA and indicated in Fig. 3, panel a. Mutagenesis data suggest that the deletion of residues 147 to 150 (ΔEYLL) and of residues 162 to 165 (ΔLKFI) highly reduces binding to RPA70 [40]; both these segments are highlighted in red in Fig. 3, panel b. Furthermore, deletion of residues 168 to 171 (ΔKNPH) reduces binding only moderately, while deletion of 157 to 160 (ΔKREP) does not affect binding [40]. Considering the location of these segments within the XPA fold it is conceivable that some of the deletions affect binding to RPA70 due to the changes they may induced in the protein secondary structure and not necessarily because these patches constitute a protein–protein docking site [40]. Nevertheless, NMR data suggest that the RPA70 protein binding surface involved in DNA binding is also implicated in XPA binding [64]. All these information together support the structure of a ternary XPA_98–219_–DNA–RPA70 complex where one strand of *ss*DNA channels through the two proteins, interacting with both the XPA_98–219_ and RPA70. This hypothesis is also in agreement with kinetic data showing that the XPA–RPA complex is 2.5 fold faster than RPA alone for binding a duplex cisplatin-damaged DNA [56]. Protein–protein docking performed with the online tool ClusPro [65], [66] using the structures of XPA and of the *ss*DNA–RPA70 complex available in the PDB with PDBids 1XPA and 1JMC, respectively, returns as the highest scoring pose a quite interesting model for a possible three-body XPA_98–219_–DNA–RPA70 complex. This complex, shown in Fig. 4, sees the *ss*DNA channeling within a cavity formed between XPA_98–219_ and RPA70. This model predicts that two poorly structured XPA_98–219_ regions, one located between residues 98 and 138, which includes the Zn coordination site, and the other located between residues 179 and 183, are also involved in the interaction with RPA70 and *ss*DNA, leaving the helical packing motif outfacing the *ss*DNA–RPA70 interface. Interestingly, as shown in Fig. 4, panels a and b, in agreement with the available experimental data [17], [45], [55], the ClusPro model suggests direct interactions with RPA70 for residues K179 and K167 and with the *ss*DNA for residue K168. The position of the XPA_98–239_ core at the 3′ side of the Y DNA junction, as shown in Fig. 4, panel c, satisfies the requirement that residues in the XPA α helix 1 are also involved in DNA binding [44] together with the potential fourth α helix (α helix 4) between residues 200 and 228 discussed above [15]. Within this hypothesis, the extended XPA_98–239_ core would bind both *ss*DNA branches at the junction, with the damaged oligonucleotide interacting with α helix 1 (and with the hypothetical α helix 4) and the undamaged branch going through the XPA_98–239_–RPA70 interface, as predicted by the ClusPro docking model.

## 5 Module 2: The XPA_29–46_–RPA32 complex

**Fig. 5:**
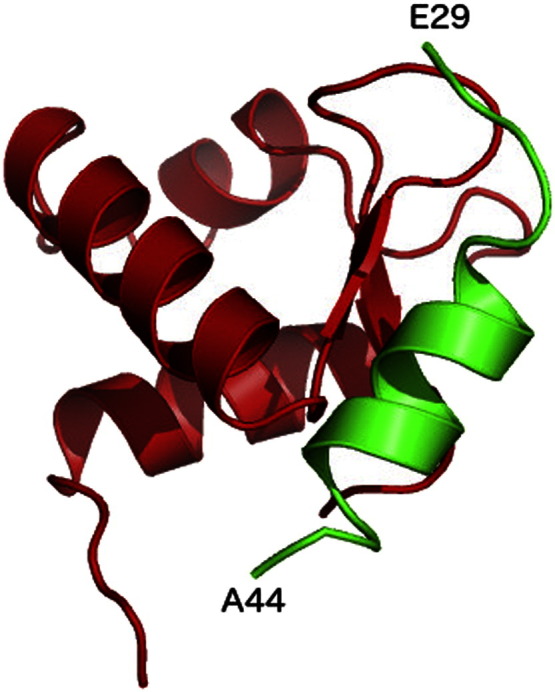
Model of the complex between the C terminal domain of RPA32 and the XPA_29–44_ peptide. This model was built based on the complex between the C terminal domain of RPA32 and the UNG_73–88_ peptide with PDBid 1DPU, where the UNG_73–88_ residues have been mutated according to the sequence alignment with the XPA_29–44_ stretch.

In the NER pre-incision complex XPA interacts also with the other large unit of RPA, namely RPA32 [17], [29], [67]. As shown in Fig. 2, the RPA32 binding motif was mapped between residues 29 and 46 in the poorly structured N terminal tail of XPA [29]. This motif binds a globular domain located in the C-terminus of RPA32, between residues 204 and 270, a docking point also shared by UNG2 and RAD52 [29]. The *ss*DNA binding region of RPA32 is located in an unstructured region of the protein, between residues 43 and 171 [29], [68]. Although the binding affinity of this region alone for the *ss*DNA is moderate [69], the binding affinity of the whole RPA heterotrimer is high, around 5 × 10^− 8^ M [29], [68], [69], [70], [71], suggesting RPA as a possible anchoring point for XPA on the DNA bubble. Furthermore, the full length XPA–RPA dissociation constant (*k_D_*), obtained by surface plasmon resonance (SPR), is in the order of 2 × 10^− 8^ M, showing a higher affinity of XPA for RPA, relative to ERCC1 [67] (see next section for details). Although a solution structure of the XPA_29–46_ peptide bound to RPA32 is not available, ^15^N-HSQC data indicate that the binding mode and the binding site of the XPA_29–46_–RPA32 complex is identical to the one characterized for the UNG_73–88_–RPA32 complex, shown in Fig. 5, where the UNG_73–88_ peptide, disordered in solution, adopts a helical structure upon binding [29]. As shown in Fig. 2, secondary structure predictions obtained with the sD2 [61] and PsiPred [62] methods indicate a propensity for helical motifs corresponding to the XPA_29–46_ sequence, while the disorder prediction method DisEMBL [63] does not flag this region as disordered.

## 6 Module 3: The XPA_67–80_–ERCC1–XPF complex

The ERCC1–XPF is a heterodimeric endonuclease responsible for cleaving the damaged *ss*DNA oligonucleotide at the 5′ side of the lesion. The nuclease activity resides entirely on the XPF module [72], while the ERCC1 is responsible for binding both, the *ss/ds*DNA Y junction and XPA [26], [27], [54], [73]. XPA is responsible for recruiting the ERCC1-XPF endonuclease to the damage site [59]. Inhibition of the XPA interaction with ERCC1–XPF blocks NER [54], [58], [59]. As shown in Fig. 2, the minimum ERCC1 binding motif of XPA is 14 residue long and it is located in a poorly structured region of the XPA N terminal tail, namely between residues 67 and 80 [54]. A peptide with sequence corresponding to this minimum binding motif, named XPA_67–80_ peptide, binds specifically the ERCC1 central domain (cERCC1), comprising residues 96 to 214, with submicromolar affinity. The XPA_67–80_ peptide binding inhibits the interaction with the full length XPA and blocks NER progression, without affecting nuclease activity [54]. The XPA_67–80_ sequence is highly conserved in all species carrying NER genes [54], [74], [75], suggesting important structural and functional roles for the 14 residues. Molecular dynamics (MD) simulation studies of the wild type and selected mutants XPA_67–80_ peptides bound to cERCC1 and free in solution suggest that, while specific residues, such as Asp 70, Phe 75 and Ile 76, are involved in direct interactions with the cERCC1 binding site, other residues affect the peptide conformational propensity while free (unbound) in solution, thus its recognition by cERCC1 [74], [75]. More specifically, while NMR data show that the unbound XPA_67–80_ peptide is poorly structured [54], extensive MD simulations, scanning the microsecond time scale, allowed to identify a degree of order within the longer timescale disorder of the XPA_67–80_ peptide [74], [75]. Indeed the wild type peptide shows a distinct conformational propensity for hairpin structures in solution, where these hairpins are structurally similar to the cERCC1-bound XPA_67–80_ conformation [54], and are stable at the low microsecond time scale [74], [75].

**Fig. 6:**
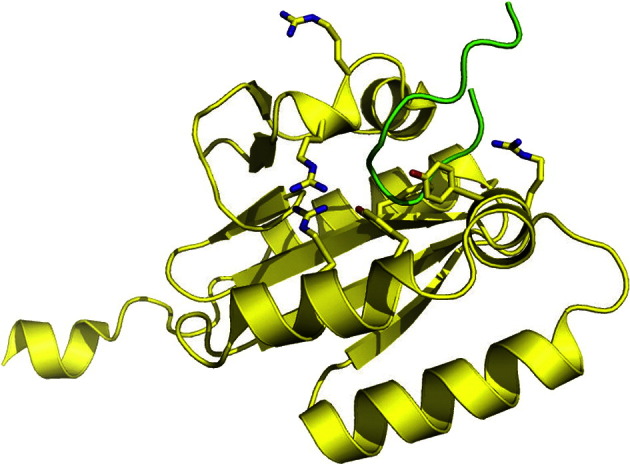
Structure of the ERCC1 central domain (PDBid 2A1I), shown in yellow, bound to the XPA_67–80_ peptide (PDBid 2JNW), shown in green. Residues implicated in interaction with the *ss*DNA are highlighted with sticks [27].

The XPA binding site of cERCC1, shown in Fig. 6, is a narrow, V-shaped, hydrophobic pocket [27], [73]. Structural comparison of the cERCC1 to the nuclease domain of the Hef nuclease indicates that the same V-shaped groove could mediate *ss*DNA-binding activity [27]. While the XPA_67–80_ peptide has been shown to be a competitive inhibitor of *ss*DNA binding by cERCC1 [54], chemical shift perturbation experiments have shown that the both full-length XPA and *ss*DNA can bind the cERCC1 simultaneously, with the cERCC1 in contact with 3 or 4 unpaired bases at most [73]. The *k_D_* of the full length XPA–cERCC1 complex from SPR, is 2.5 × 10^− 7^ M [67], while the value of 2.5 × 10^− 6^ M was obtained for the *k_D_* of the cERCC1 in complex with a 10 unpaired nucleotide-long DNA bubble [73].

## 7 Proposed role for XPA in the NER pre-incision complex assembly

The intricacy and high specificity of the XPA–protein and XPA–*ss*DNA interactions is in apparent contrast with the lack of structure characterizing large part of the XPA sequence and with the short length of some of the XPA interaction domains. This high degree of conformational disorder is consistent with the lack of structural information we have on the full-length XPA, when bound and especially when unbound in solution. Within the whole XPA sequence, shown in Fig. 2, we can identify different degrees of conformational propensity, from the highly disordered to structured. In some instances structured and partially structured regions correspond to protein–protein and protein–DNA interaction hubs, such as the XPA_98–239_ domain, specific for binding *ss*DNA and RPA70. In the cases of the poorly structured XPA_29–46_ and XPA_67–80_ domains, specific to RPA32 and cERCC1, respectively, secondary structure motifs may go undetected when the protein is free in solution due to the length of experimental timescales. For example, in the case of XPA_67–80_, a specific hairpin motif structurally similar to the bound conformation has been found to be significantly populated at the low microsecond time scale [74], [75], when it can be selectively recognized and bound by cERCC1. As a support for conformational selection [75], [76], [77], [78] as a recognition mechanism, simulation data [75] show that the XPA_67–80_ conformational propensity in solution can be significantly affected by the mutation of the terminal residues of the cERCC1-specific region, namely Lys 67 or Glu 78 to 80, among others, where the mutants show a much higher level of structural disorder relative to the wild type [75]. This significant increase in structural disorder can explain the inability to bind cERCC1 of a XPA mutant where the stretch between Glu 78 and Glu 84, termed E motif, was deleted [58], [75].

While the XPA–protein and XPA–*ss*DNA binding regions fall in a range between highly (XPA_98–219_) to less structured (XPA_29–46_ and XPA_67–80_), where the observation timescale defines the position within this range, the linker regions connecting the different XPA binding motifs are intrinsically disordered. The presence of these structureless regions confers to XPA the ability to function as a flexible scaffold with a “bead on a string” architecture, avoiding major steric clashes in the organization of the NER core proteins around the DNA bubble framework [6]. Furthermore, structural flexibility represents an advantage not only in terms of steric constraints, but also because it modulates the binding affinity, thus it allows for sequential binding [6], [79]. The higher the structural disorder of a molecule unbound in solution, the higher the entropic penalty to pay when that conformational freedom is lost upon binding. Thus, the high conformational flexibility characterizing the XPA_67–80_ region confers a lower binding affinity to the ERCC1–XPA_67–80_ complex relative to the XPA_29–46_–RPA32 complex and especially to the structured XPA_98–219_–*ss*DNA-RPA70 complex [54], [67]. Indeed, according to secondary structure predictions shown in Fig. 2, the XPA_29–46_ region has an intrinsic propensity to form helices, which makes XPA_29–46_ less disordered than the XPA_67–80_ region.

Based on the data available and on the sequential binding discussed above, the following role for XPA in the pre-incision complex modular architecture is proposed and represented schematically in Fig. 1, panels c and d. Although there is evidence supporting the participation of XPA in damage recognition [33], possibly as a homodimer [34], [35], [36], the mainstream current understanding suggests that the recruitment and positioning of XPA to the damage site could take place *via* its interaction with either XPC–RAD23B [41], or TFIIH [42], post lesion recognition by XPC–RAD23B. The XPA main anchoring point around the DNA bubble is most likely the heterotrimeric RPA complex, that has a high binding affinity for both, damaged *ss*DNA [67], [70], [80] and XPA [67]. The (mostly) structured XPA_98–239_ core binds RPA70 and the *ss*DNA in a ternary complex that constitutes the first module (Module 1) in the pre-incision bubble. Although there are no direct structural information on the XPA_98–239_–*ss*DNA-RPA70 complex, the available data, discussed in detail in a previous sections, support a structure where the *ss*DNA channels through an interface formed between the RPA70 and the XPA_98–239_, located at the 3′ side of the lesion. A model for the Module 1 complex that fits with the available experimental evidence was generated through protein–protein docking with the online tool ClusPro [65], [66] and it is shown in Fig. 4. The other large domain of RPA, namely RPA32, constitutes the second anchoring point for XPA around the DNA bubble (Module 2). RPA32 binds the *ss*DNA alongside the RPA70 on the damaged oligonucleotide. The N-terminal XPA_29–46_ stretch binds specifically a globular domain in the RPA32 C-terminus [29]. As shown in Fig. 2, the two RPA-binding domains of XPA are linked though a largely disordered region, counting over 50 residues. Roughly central within this region is the cERCC1-binding sequence. As a possible mechanism for the assembly of the pre-incision complex, the recruitment and positioning of the ERCC1–XPF endonuclease by XPA depends on the formation of Modules 1 and 2 as anchoring points. Indeed, the poorly structured 50 residue linker, connecting the XPA–*ss*DNA–RPA70 and XPA–RPA32 units, constitutes a loop wide enough to reach the Y junction at the 5′ side, located approximately 30 nucleotides away, thus to bind and position the ERCC1–XPF endonuclease. Once the ERCC1–XPF endonuclease is put in place, it starts the dual incision step followed by the XPG nick at the 3′ side [81], which results in the elimination of the damaged oligonucleotide.

## 8 Summary and concluding remarks

The conformational flexibility conferred to proteins by structural disorder can provide many functional advantages over highly structured domains [1], [2], [3], [4], [6]. These advantages are fully exploited by higher eukaryotes, with over 40% of proteins coded by the human genome containing structurally disordered regions [3], [5]. Here I have reviewed and discussed, in view of the available experimental and computational data, the structure and function relationship and the role of structural disorder in XPA, a scaffolding protein essential for the progression of the NER pathway [18]. The analysis presented here shows that XPA exerts its role as a scaffold through the formation of 3 main interactions modules. Module 1 is a heterotrimeric complex involving the XPA_98–239_ region, the *ss*/*ds*DNA Y junction at the 3′ side of the lesion and RPA70, likely to function as the main anchoring point for XPA on the DNA bubble [67]. A model of this ternary complex was also presented, which may provide new insight for the design of mutagenesis studies. The N terminal XPA_29–46_ region, namely binds the C-terminal globular domain of RPA32 [29], to form Module 2, most likely through a conformational selection mechanism. Conformational selection also plays a role in the formation of Module 3, which involves a 14 residue section of the least structured region of XPA, namely XPA_67–80_, and the central domain of ERCC1 (cERCC1) [74], [75]. XPA_67–80_ is located in the middle of a 50 residue-long intrinsically disordered loop, connecting Modules 1 and 2. This loop is wide and flexible enough to reach the Y DNA junction at the 5′ side of the lesion and to position the ERCC1-XPF endonuclease for the dual excision. The XPA_67–80_–cERCC1 interaction has the lowest binding affinity between the 3 [67], thus it is likely to occur last. This analysis shows that the different degrees of structural disorder in the XPA protein allow it to adopt a “beads on a string” architecture, ideal to fit within the DNA bubble framework, avoiding steric clashes. Furthermore, because of the thermodynamic interplay between enthalpic and entropic contributions, the balance between order and disorder has a significant effect on the relative binding affinities of the modules, allowing for their sequential (and possibly reversible) assembly. In conclusion, with conformational selection playing a huge role in the molecular recognition and binding of poorly structured protein domains [6], [75], [76], [77], [78], this perspective on the XPA protein contributes to highlight that the concept of structural order and disorder becomes highly dependent on the timescale difference between the experimental measurement and the molecular recognition and that it cannot be considered as an absolute observable. Because of the key role played by XPA in NER, an interesting point for further investigation into its structure–function relationship is the effect of single nucleotide polymorphisms (SNPs) and of non-frameshifting insertion and deletions (INDELs), commonly found in disordered linking regions [82]. Indeed, SNPs in DNA repair proteins seem to be related to DNA repair abilities, cancer risk [83] and chemotherapeutic resistance [84]. The frequency, nature and lengths of INDELs in the XPA linking regions may affect its effectiveness as a flexible scaffold, thus the overall function in NER pathway, conferring a distinctive genetic trait for disease predisposition or therapeutic resistance.

## Figures and Tables

**Table 1:** NER core factors involved in the lesion recognition (GG-NER) and excision with corresponding molecular weights, function, and main interactions with other NER proteins.

NER factors	Molecular weight (kDa)	Core NER factor interactions	NER function
XPC–RAD23B	106 + 43	XPA, TFIIH	Damage recognition and NER factors recruitment
RPA	70 + 32 + 14	XPA, XPG	Pre-incision complex anchor and NER factors recruitment
XPA	40	ERCC1, RPA, TFIIH, XPC-RAD23B	Pre-incision complex scaffold and NER factors recruitment
TFIIH	460	XPA, XPC, XPG	Damage recognition, helicase and NER factors recruitment
XPG	133	RPA, TFIIH	3′ endonuclease
ERCC1–XPF	38 + 112	XPA	5′ endonuclease
